# Supplementary material for: Determining the optimal number of independent components for reproducible transcriptomic data analysis
Source: BMC Genomics. 2017 Sep 11;18:712. doi: 10.1186/s12864-017-4112-9 (PMC5594474; doi:10.1186/s12864-017-4112-9)

METABRIC dataset

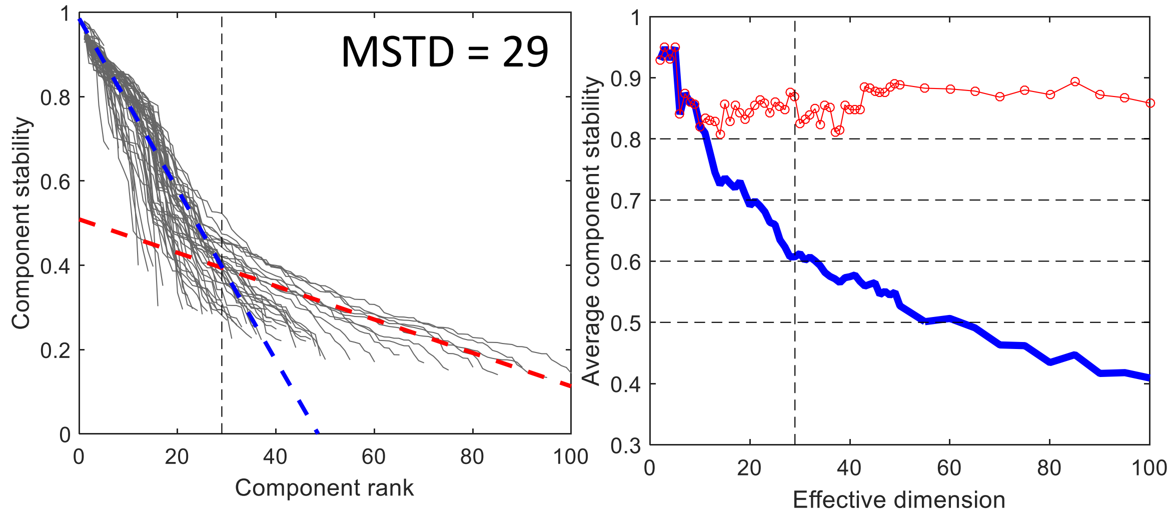

BRAC TCGA dataset

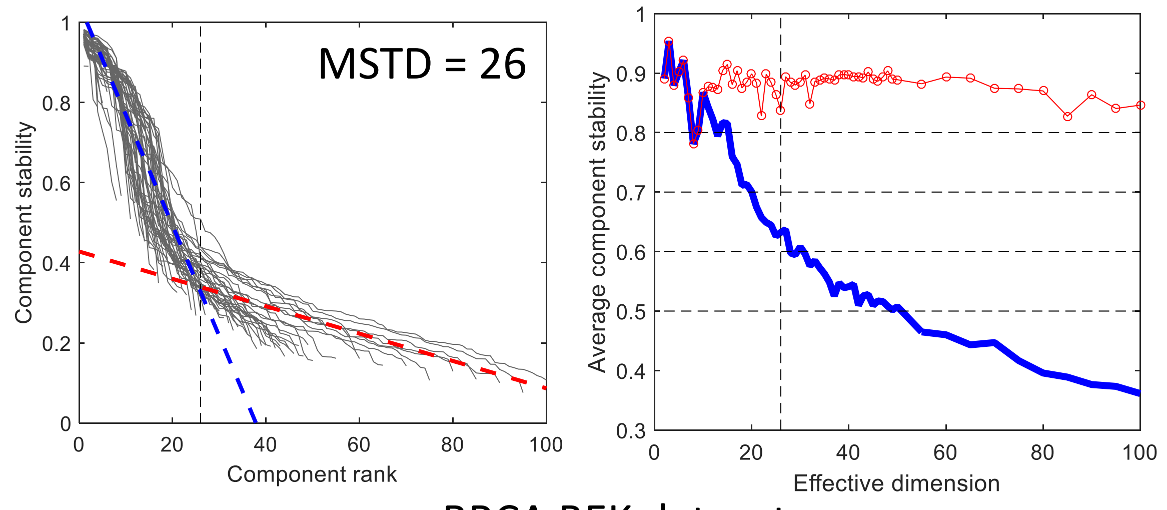

BRCA CIT dataset

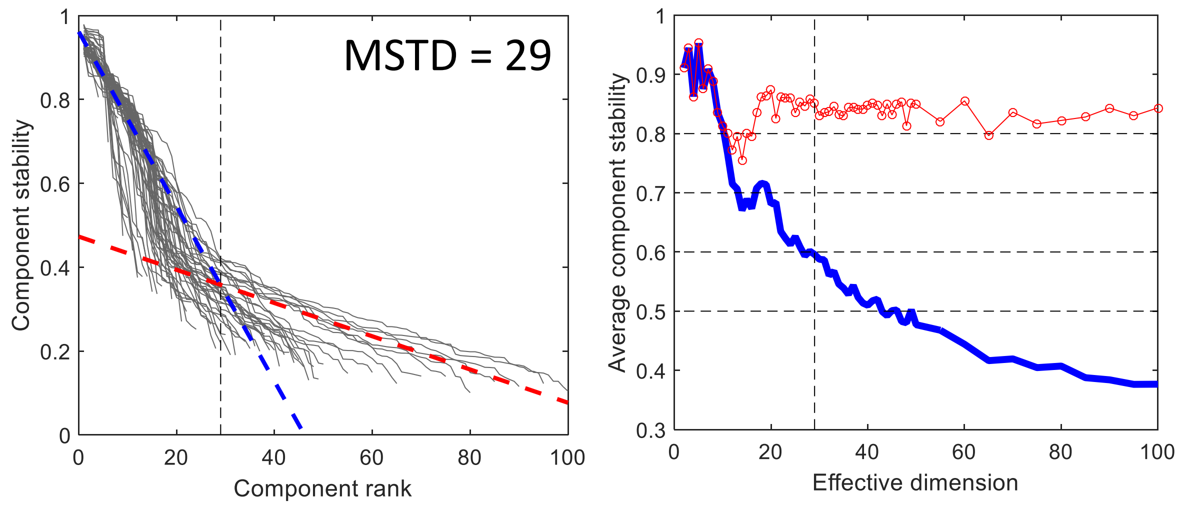

BRCA BEK dataset

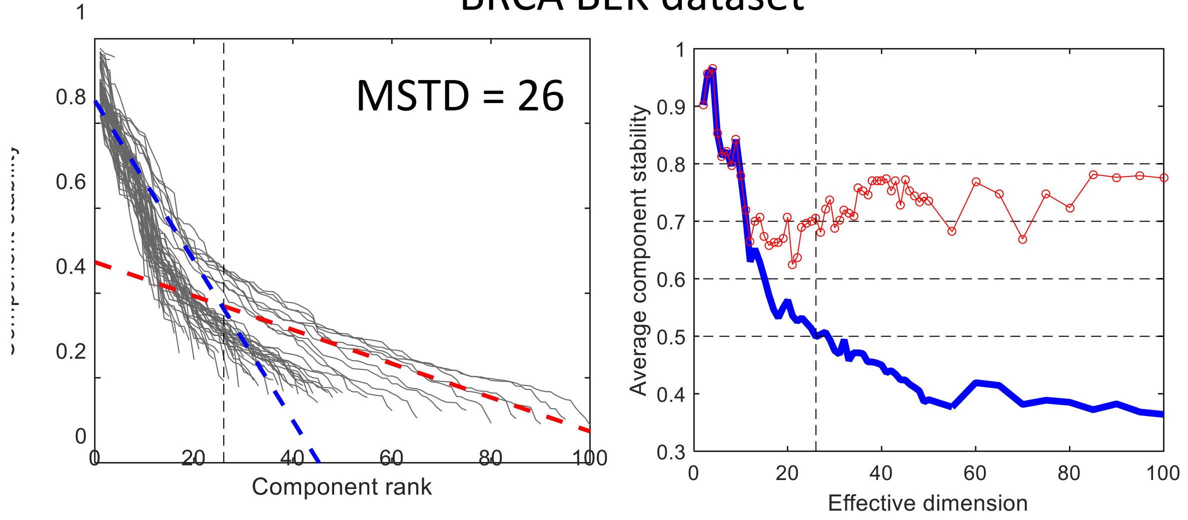

BRCA BCR dataset

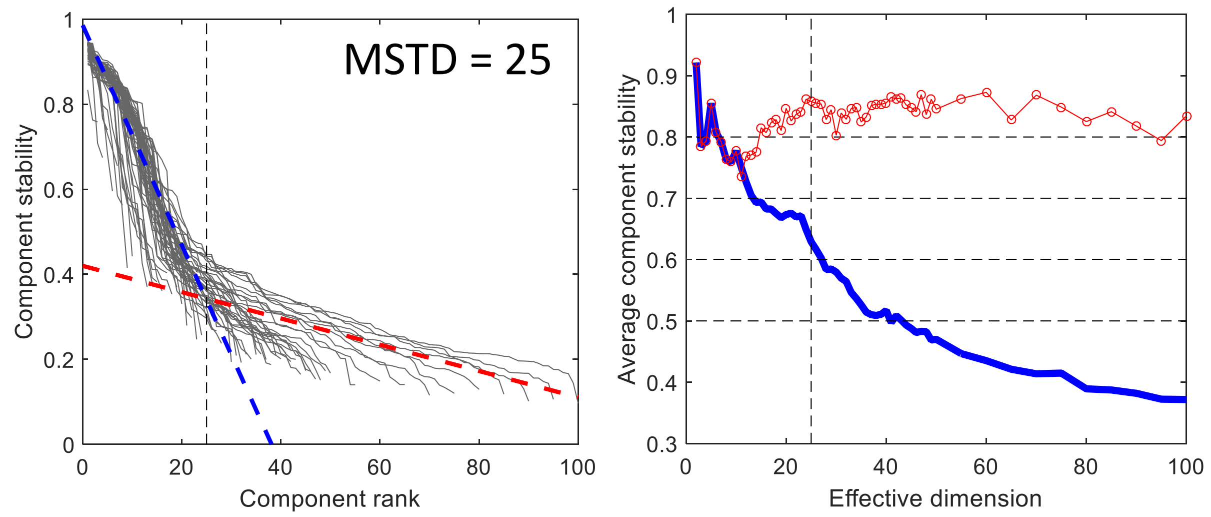

BRCA WANG dataset

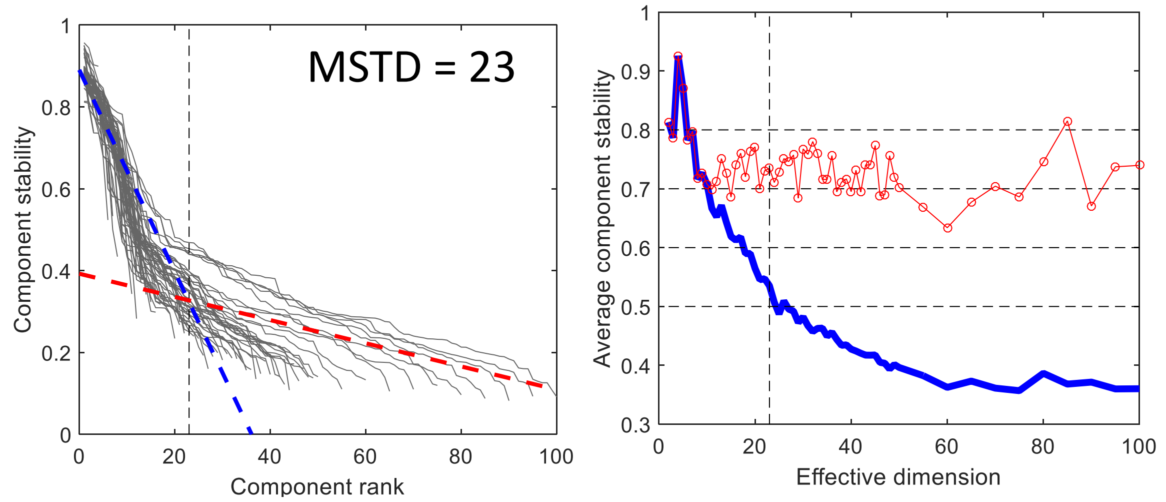

Supplement: Supplementary file 1 — Estimating MSTD dimension for six breast cancer datasets. The notations are the same as in Fig. 1. (PDF 479 kb) [file 12864_2017_4112_MOESM1_ESM.pdf]
