# Supplementary figures and images for: Determining the optimal number of independent components for reproducible transcriptomic data analysis
Source: BMC Genomics. 2017 Sep 11;18:712. doi: 10.1186/s12864-017-4112-9 (PMC5594474; doi:10.1186/s12864-017-4112-9)

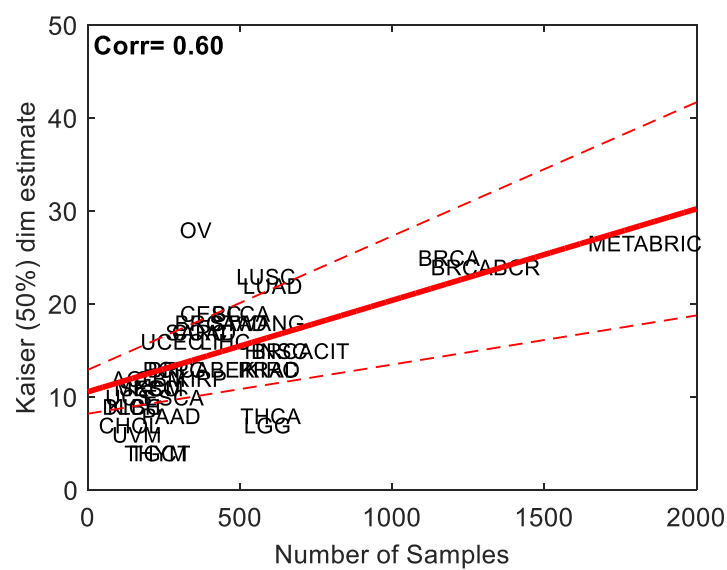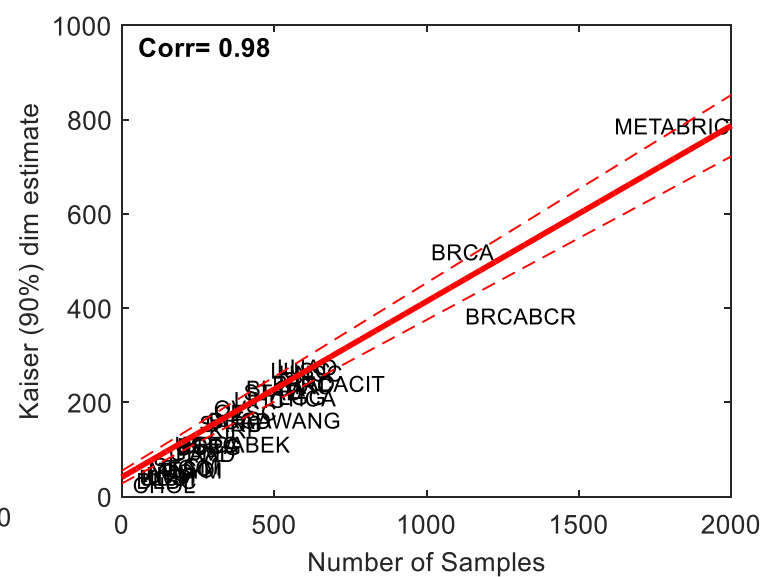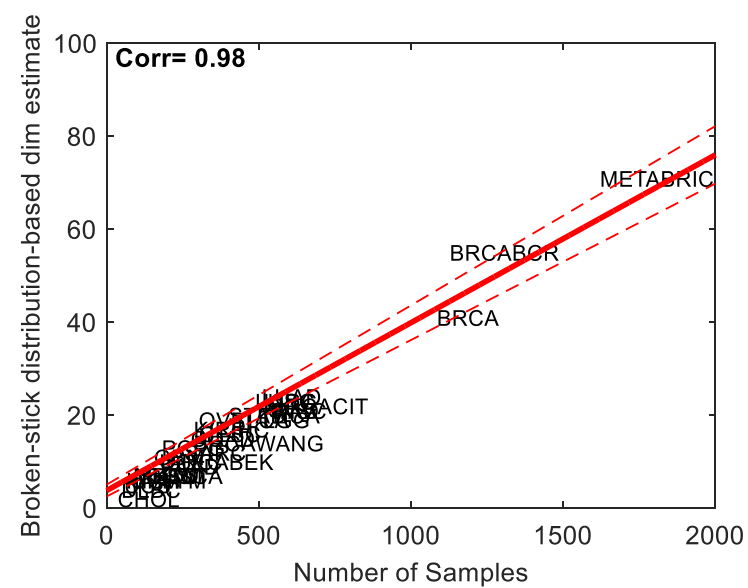

Supplement: Supplementary file 2 — Standard estimations of intrinsic dimensionality (by Keiser rule or by broken stick distribution) of cancer datasets. (PDF 288 kb) [file 12864_2017_4112_MOESM2_ESM.pdf]

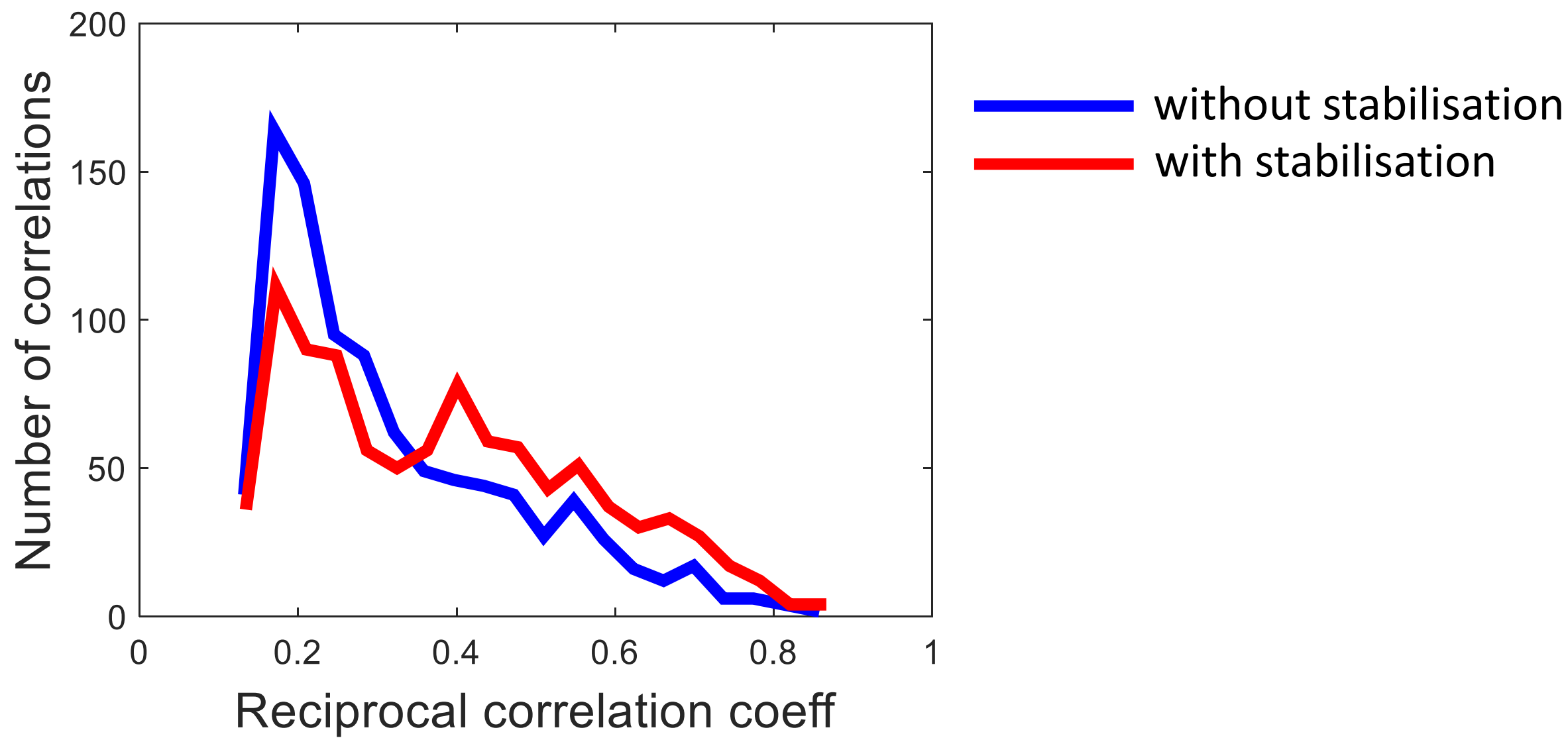

Supplement: Supplementary file 5 — The histograms of the total number of reciprocal correlations in the correlation graph such as the one shown in Fig. 3, with and without applying the component stabilization approach. (PDF 164 kb) [file 12864_2017_4112_MOESM5_ESM.pdf]

Time to compute 100 runs of fastICA

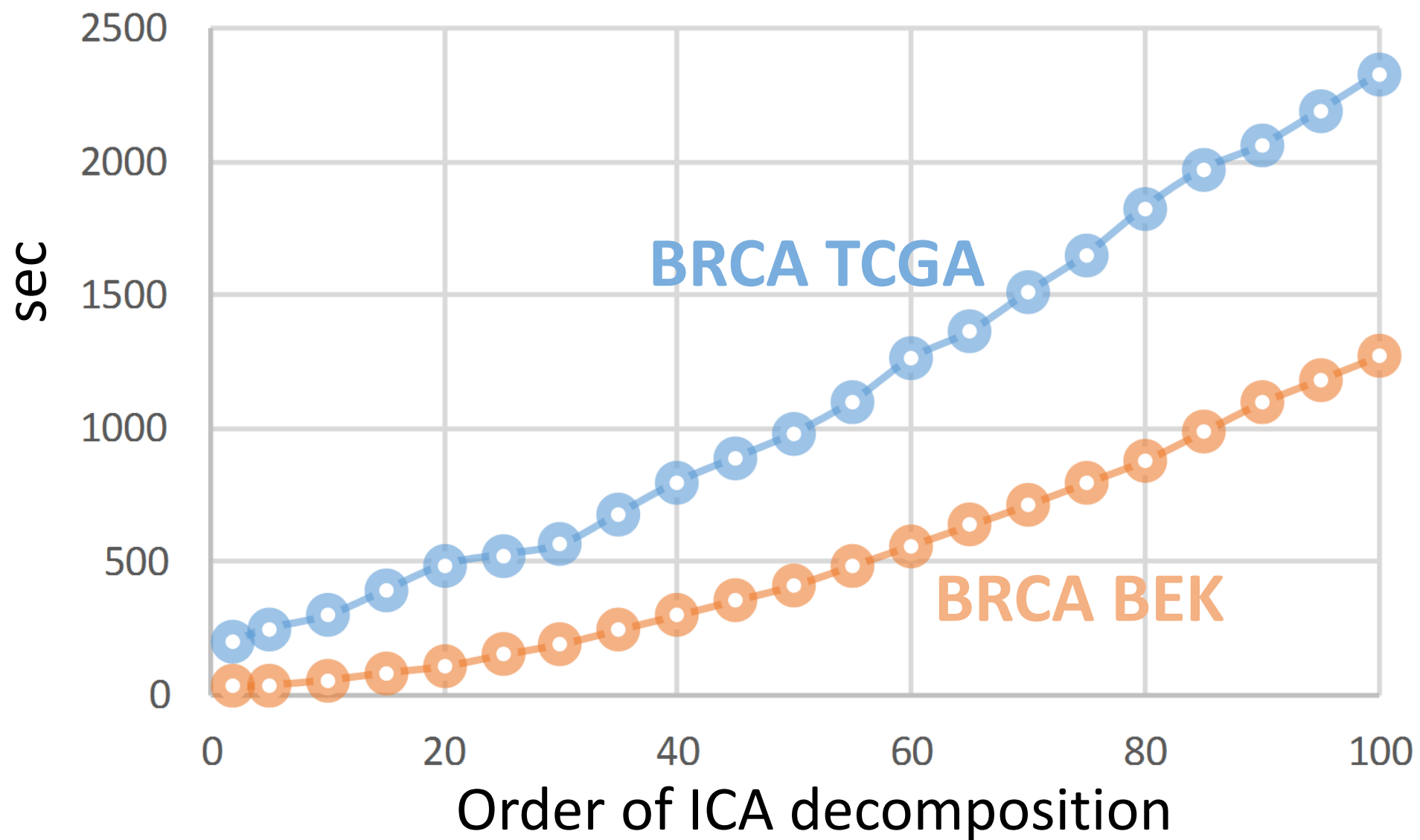

Supplement: Supplementary file 6 — Computational time for ICA decomposition of different orders from 2 to 100 with step 5, using compiled MATLAB fastICA implementation and stability analysis by re-computing fastICA from 100 various initial conditions. The computation is made using an ordinary laptop with Intel Core i7 processor and 16Gb of memory, in a single thread. The BRCA BEK dataset (from [27]) contains 10,000 genes in 197 samples, and the BRCA TCGA dataset (from [28]) contains 20,503 genes in 1095 samples. The overall timing for computing all ICA decomposition with their stability analysis is 3.0 h for BRCA BEK dataset, and 6.5 h for BRCA TCGA dataset. These computations can be repeated using BIODICA software [29] (https://github.com/LabBandSB/BIODICA), by launching ICA computation in scanning mode. (PDF 361 kb) [file 12864_2017_4112_MOESM6_ESM.pdf]

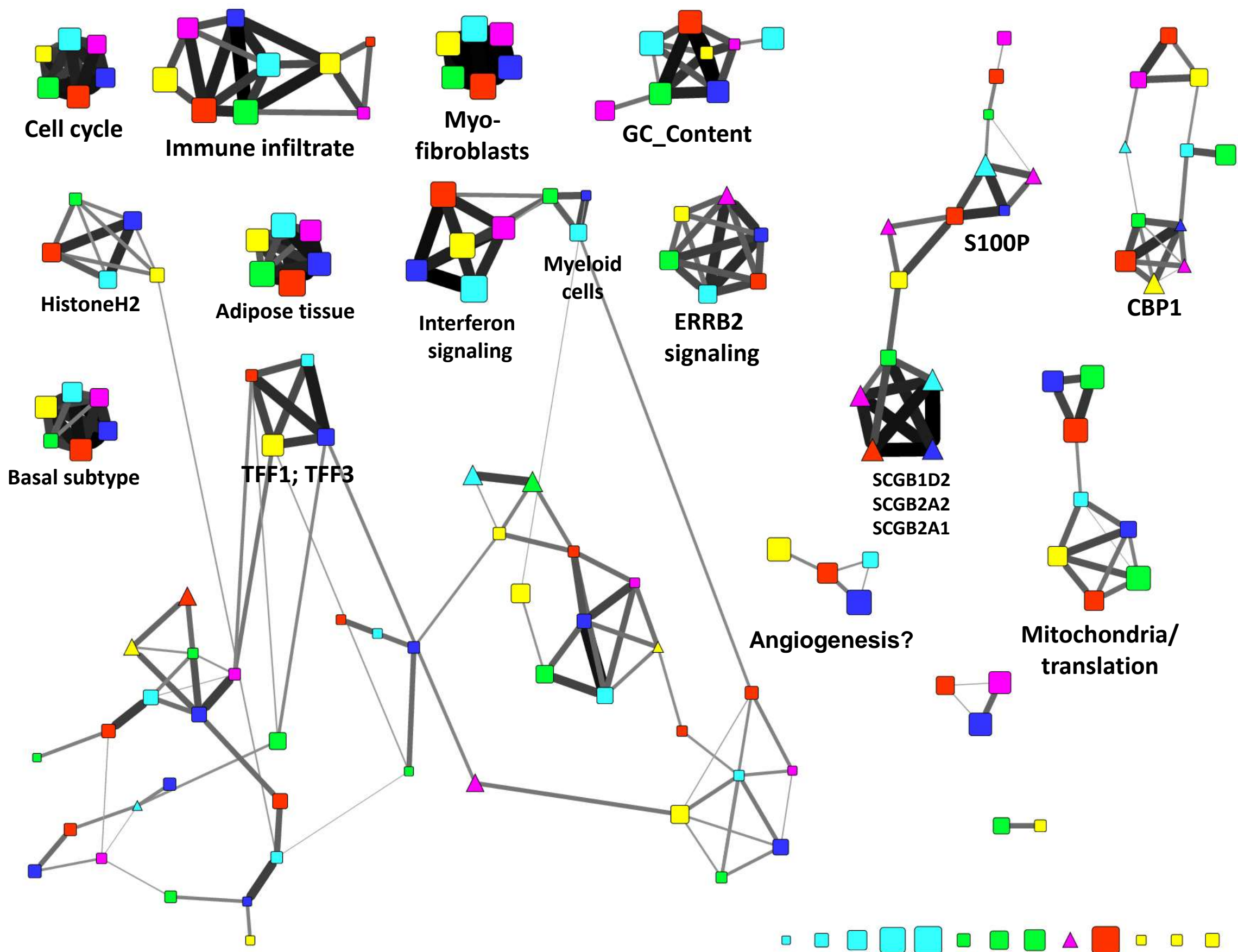

Supplement: Supplementary file 7 — Graph of reciprocal correlations between components computed with MSTD choice for the reduced dimension and the number of components. The size of the points reflects their stability (larger points corresponds to more stable components). The color and the width of the edges reflect the Pearson correlation coefficient. Propositions of annotations of the pseudo-cliques in the graph are made based on the comparison with previously annotated metagenes [3] and the analysis of the top contributing genes using hypergeometric test and the toppgene web tool [30]. (PDF 315 kb) [file 12864_2017_4112_MOESM7_ESM.pdf]
